# ﻿Diversity of *Distoseptispora* (Distoseptisporaceae) taxa on submerged decaying wood from the Red River in Yunnan, China

**DOI:** 10.3897/mycokeys.102.116096

**Published:** 2024-02-05

**Authors:** Hong-Wei Shen, Dan-Feng Bao, Saranyaphat Boonmee, Yong-Zhong Lu, Xi-Jun Su, Yun-Xia Li, Zong-Long Luo

**Affiliations:** 1 College of Agriculture and Biological Science, Dali University, Dali 671003, Yunnan, China; 2 Center of Excellence in Fungal Research, Mae Fah Luang University, Chiang Rai 57100, Thailand; 3 School of Science, Mae Fah Luang University, Chiang Rai 57100, Thailand; 4 School of Food and Pharmaceutical Engineering, Guizhou Institute of Technology, Guiyang 550003, Guizhou, China

**Keywords:** 2 new taxa, lignicolous freshwater fungi, phylogeny, Sordariomycetes, taxonomy

## Abstract

The Red River Basin is located in the Indo-Burma biodiversity hotspot and is rich in lignicolous freshwater fungi, but no systematic research has been conducted. A systematic study on the species diversity of lignicolous freshwater fungi in the basin is ongoing. Seven distoseptispora-like specimens were collected from the Red River Basin in Yunnan. Phylogenetic analysis of ITS, LSU, *tef*1-α, and *rpb*2 genes and combined morphological data indicate that there are six distinct species of *Distoseptispora*, including two new species and four known species. Two new species were named *D.suae* and *D.xinpingensis*, and the four known species were *D.bambusae*, *D.euseptata*, *D.obpyriformis* and *D.pachyconidia*. This study provides detailed descriptions and illustrations of these six species and an updated phylogenetic backbone tree of *Distoseptispora*.

## ﻿Introduction

The present study is to establish the species of freshwater fungi along a north-to-south longitudinal gradient (Hyde et al. 2016). Yunnan is one of the hotspots for lignicolous freshwater fungi, where numerous species have been reported (Su et al. 2016; Luo et al. 2017, 2018a, b, 2019; Bao et al. 2020; Dong et al. 2020, 2021). In Yunnan, 278 lignicolous freshwater fungi have been identified in both lentic habitats (Cai et al. 2002; Luo et al. 2004) and lotic habitats, including the Nu Jiang/Salween River, Lancang River/Mekong River, Dulong River, and Jinsha River/Yangtze River (Tsui et al. 2000; Luo et al. 2018a, b, 2019; Bao et al. 2020, 2021; Dong et al. 2020, 2021; Shen et al. 2022). However, the species diversity and distribution of lignicolous freshwater fungi in the Red River Basin remain under-explored.

The Red River, one of the largest rivers in Southeast Asia, originates in Weishan County, Dali Bai Autonomous Prefecture, Yunnan Province, China, it has a total length of 1200 km with a catchment area of 169,000 km^2^ (Haruyama 1995; Van den Bergh et al. 2007). Of this area, 50.3% is in Vietnam, 48.8% in China, and 0.9% in Laos (Van Maren 2007). The portion of the Red River Basin located in China is referred to as “Yuanjiang”. This segment has a length of 677 km and is characterized by a plateau monsoon climate (Gu et al. 2018). Precipitation in the basin generally decreases from downstream to upstream and increases from the valleys to the mountains. The basin boasts a wealth of biological resources (Jiang 1980; Zhou and Cui 1997; Chen and Yu 2013; Gu et al. 2018).

*Distoseptispora* is a well-studied phylogenetic genus introduced by Su et al. (2016) to accommodate some *Sporidesmium* taxa with unbranched, olive green, cylindrical conidiophores, monoblastic, integrated, determinate, terminal, cylindrical conidiogenous cells, and acrogenous, distoseptate, cylindrical, smooth, darker conidia with slightly paler (but not hyaline), rounded apices of indeterminate length (Su et al. 2016; Yang et al. 2018, 2021; Zhang et al. 2022). Based on morphological and phylogenetic analyses, 73 species have been accepted in *Distoseptispora* in recent years (http://www.indexfungorum.org/Names/Names.asp; accessed on 2 January 2024; Hu et al. 2023). These include the type species *D.fluminicola* McKenzie, Hong Y. Su, Z.L. Luo & K.D. Hyde and two species transferred from *Ellisembia* as *D.adscendens* (Berk.) R. Zhu & H. Zhang and *D.leonensis* (M.B. Ellis) R. Zhu & H. Zhang. Among them, *D.hyalina* and *D.licualae* are the only two teleomorph taxa in *Distoseptispora* (Yang et al. 2021; Konta et al. 2023). Members of *Distoseptispora* are primarily saprophytes found on woody substrates from freshwater habitats (45 species), predominantly in China and Thailand and some also have been found in terrestrial habitats (23 species); *D.bambusae*, *D.clematidis*, *D.tectonae*, *D.thysanolaenae* and *D.xishuangbannaensis* have been reported in both freshwater and terrestrial habitats (Hyde et al. 2016; Luo et al. 2018a, 2019; Yang et al. 2018, 2021; Monkai et al. 2020; Phukhamsakda et al. 2020; Sun et al. 2020; Dong et al. 2021; Li et al. 2021; Shen et al. 2021; Ma et al. 2022; Zhai et al. 2022; Zhang et al. 2022). The conidia of *Distoseptispora* vary significantly in their characteristics, especially in terms of shape and size. Zhang et al. (2022) reassessed both the generic and specific boundaries of *Distoseptispora*, and summarized the characteristics of species in this genus, encompassing various attributes such as the length of conidiophores, proliferation, and conidiogenesis in conidiogenous cells, and details about conidia, including their type (distoseptate or euseptate), number of septa, shape, length, color, proliferation, rostrate nature, and wall thickness. Even though *Distoseptispora* species formed three distinct clades in phylogenetic analysis results that received strong support, the morphological characters of these species (such as monoblastic/polyblastic, euseptate/distoseptate) only offer species-level differentiation and do not hold taxonomic significance for the broader categorization of *Distoseptispora* (Yang et al. 2021; Zhang et al. 2022).

A systematic investigation of lignicolous freshwater fungal diversity and distribution in the Red River Basin is ongoing. This study represents the first report of *Distoseptispora* species in the Red River Basin. A morphological examination combined with phylogenetic analysis combining internal transcribed spacer (ITS), large subunit nuclear ribosomal RNA (LSU), translation elongation factor 1-alpha (*tef*1-α) and second-largest subunit of RNA polymerase II (rpb2) sequence data, established that out of seven distoseptispora-like specimens collected in the Red River Basin, six species of *Distoseptispora* were identified, including two new species, named as *D.suae* and *D.xinpingensis* and four known species, viz. *D.bambusae*, *D.euseptata*, *D.obpyriformis* and *D.pachyconidia*.

## ﻿Materials and methods

### ﻿Specimen collection, examination and isolation

Specimens of submerged decaying wood were collected from the Yuanjiang Basin (Red River) in Yunnan, China. The samples were incubated in a plastic box at room temperature for one week. Morphological observations were conducted following the methods of Luo et al. (2018a) and Senanayake et al. (2020) with a few modifications. Macromorphological characteristics of the samples were observed using an Optec SZ 760 compound stereomicroscope (Chongqing Optec Instrument Co., Ltd, Chongqing, China). Preliminary microscope slides were examined and photographed under a Nikon ECLIPSE Ni-U compound stereomicroscope (Nikon, Tokyo, Japan). Colonies’ morphologies on native substrates were captured using a Nikon SMZ1000 stereo-zoom microscope (Nikon, Tokyo, Japan). The measurements of photomicrographs were obtained using Tarosoft (R) Image Frame Work version 0.9.7. Images were edited with Adobe Photoshop CS5 Extended version 12.0.0.0 software (Adobe Systems, San Jose, CA, USA).

Single spore isolations were carried out based on the method described by Luo et al. (2018a). The individually germinated conidia were transferred to fresh potato dextrose agar (PDA, from Beijing Bridge Technology Co., Ltd., Beijing, China) plates and incubated at room temperature in the dark. Some of the remaining germinated spores, along with their agar, were placed on water-mounted glass slides to photograph the origins of the germ tubes.

After observation and isolation, specimens were air-dried naturally, wrapped in absorbent paper, and stored in a ziplock bag with mothballs. These specimens were then deposited in the herbarium of Cryptogams, Kunming Institute of Botany, Chinese Academy of Sciences (**KUN-HKAS**), Kunming, China. The cultures were deposited with the China General Microbiological Culture Collection Center (**CGMCC**), and Kunming Institute of Botany Culture Collection (**KUNCC**). Fungal Names numbers are registered in the Fungal Names database (https://nmdc.cn/fungalnames/registe; accessed on 4 August 2023; Wang et al. 2023) and Facesoffungi numbers were obtained as described in Jayasiri et al. (2015).

### ﻿DNA extraction, PCR amplification and sequencing

DNA extraction, PCR amplification, sequencing, and phylogenetic analysis were carried out following the methods described by Dissanayake et al. (2020). Mycelia used for DNA extraction were cultivated on PDA for 3–4 weeks at 24 °C. From each isolate, total genomic DNA was extracted from 100–150 mg of axenic mycelium, which was carefully scraped from the edges of the growing culture with a sterile scalpel. This material was transferred to a 1.5 mL microcentrifuge tube using sterilized inoculum needles. Mycelium was ground to a fine powder with liquid nitrogen or quartz sand to break the cells for DNA extraction. DNA was extracted with the TreliefTM Plant Genomic DNA Kit (TSP101) following manufacturer guidelines (Beijing Tsingke Biological Engineering Technology and Services Co., Ltd, Beijing, P.R. China).

Four gene regions, ITS, LSU, *tef*1-α and *rpb*2 were amplified using ITS5/ITS4 (White et al. 1990), LR0R/LR7 (Vilgalys and Hester 1990), EF1-983F/EF1-2218R (Liu et al. 1999) and RPB2-5F/RPB2-7cR (Liu et al. 1999) primer pairs, respectively. Primer sequences are available at the WASABI database at the AFTOL website (aftol.org). The PCR mixture contained 12.5 μL of 2× GS Taq PCR MasterMix (mixture of DNA Polymerase, dNTPs, Mg^2+^ and optimized buffer; Genes and Biotech, Beijing, China), 1 μL of each primer including forward primer and reverse primer (10 μM), 1 μL template DNA extract and 9.5 μL double-distilled water (Luo et al. 2018a). The PCR thermal cycling conditions were performed as presented in Table 1. PCR products were purified using mini-columns, purification resin and buffer according to the manufacturer’s protocols. The PCR sequences were carried out at Beijing Tsingke Biological Engineering Technology and Services Co., Ltd (Beijing, P.R. China).

**Table 1.** PCR thermocycling conditions for genes used in this paper.

Genes	Initial denaturation		Denaturation		Annealing		Extension		No. of cycles	Final extension	
	Temp (°C)	Time (min)	Temp (°C)	Time (s)	Temp (°C)	Time (s)	Temp (°C)	Time (s)		Temp (°C)	Time (min)
ITS	94	3	94	30	56	50	72	60	30	72	10
LSU, *tef*1-α	94	3	94	50	55	60	72	60	30	72	10
*rpb*2	94	5	94	60	52	90	72	90	38	72	10

### ﻿Phylogenetic analysis

BLAST searches using the BLASTn algorithm were performed to retrieve similar sequences from GenBank (http://www.ncbi.nlm.nih.gov, accessed on 2 January 2024) and relevant publications (Ma et al. 2022; Zhang et al. 2022; Hu et al. 2023). The sequences were aligned using MAFFT online service: multiple alignment program MAFFT v.7 (Kuraku et al. 2013; Katoh et al. 2019; http://mafft.cbrc.jp/alignment/server/index.html, accessed on 2 January 2024), and sequence trimming was performed with trimAl v1.2 with default parameters (http://trimal.cgenomics.org for specific operation steps; Capella-Gutiérrez et al. 2009). The sequence dataset was combined using SquenceMatrix v.1.7.8 (Vaidya et al. 2011). FASTA alignment formats were changed to PHYLIP and NEXUS formats by the website: ALignment Transformation EnviRonment (ALTER) (http://sing.ei.uvigo.es/ALTER/, accessed on 2 January 2024).

Maximum likelihood (ML) analysis was performed setting RAxML-HPC2 on XSEDE (8.2.12) (Stamatakis 2006; Stamatakis et al. 2008) in CIPRES Science Gateway (Miller et al. 2010; http://www.phylo.org/portal2; accessed on 25 January 2022), using the GTR+GAMMA model with 1000 bootstrap repetitions. Bayesian analyses were performed in MrBayes 3.2.6 (Ronquist et al. 2012) and the best-fit model of sequences evolution was estimated via MrModeltest 2.2 (Guindon and Gascuel 2003; Nylander 2004; Darriba et al. 2012). The Markov Chain Monte Carlo (MCMC) sampling approach was used to calculate posterior probabilities (PP) (Rannala and Yang 1996). Bayesian analyses of six simultaneous Markov chains were run for 5 M generations and trees were sampled every thousand generations.

Phylogenetic trees were visualized using FigTree v1.4.0 (http://tree.bio.ed.ac.uk/software/figtree/), editing and typesetting using Adobe Illustrator (AI) (Adobe Systems Inc., San Jose, CA, USA). The new sequences were submitted in GenBank and the strain information used in this paper is provided in Table 2.

**Table 2.** Taxa used in the phylogenetic analyses and their corresponding GenBank accession numbers.

Species	Source	GenBank accession number			
		LSU	ITS	*tef*1-α	*rpb*2
*Aquapteridosporafusiformis*	MFLUCC 18–1606^T^	MK849798	MK828652	MN194056	–
*A.lignicola*	MFLUCC 15–0377^T^	KU221018	MZ868774	MZ892980	MZ892986
*Distoseptisporaadscendens*	HKUCC 10820	DQ408561	–	–	DQ435092
*D.amniculi*	MFLUCC 17–2129^T^	MZ868761	MZ868770	–	MZ892982
*D.appendiculata*	MFLUCC 18–0259^T^	MN163023	MN163009	MN174866	–
*D.aqualignicola*	KUNCC 21–10729^T^	ON400845	OK341186	OP413480	OP413474
*D.aquamyces*	KUNCC 21–10732^T^	OK341199	OK341187	OP413482	OP413476
*D.aquatica*	MFLUCC 15–0374^T^	KU376268	MF077552	–	–
	MFLUCC 18-0646	MK849793	MK828648	–	–
*D.aquisubtropica*	GZCC 22–0075^T^	ON527941	ON527933	ON533677	ON533685
*D.atroviridis*	GZCC 20–0511^T^	MZ868763	MZ868772	MZ892978	MZ892984
	GZCC 19–0531	MZ227223	MW133915	–	–
*D.bambusae*	MFLUCC 20–0091^T^	MT232718	MT232713	MT232880	MT232881
	MFLUCC 14–0583	MT232717	MT232712	–	MT232882
	KUNCC 21–10732	OK341200	OK341188	OP413492	OP413487
***D.bambusae***	**KUNCC 22–12668**	**PP068863**	**PP068486**	**PP066113**	**PP066110**
*D.bambusicola*	GZCC 21–0667^T^	MZ474872	MZ474873	–	–
*D.bangkokensis*	MFLUCC 18–0262^T^	MZ518206	MZ518205	–	–
*D.cangshanensis*	MFLUCC 16–0970^T^	MG979761	MG979754	MG988419	–
*D.caricis*	CBS 146041^T^	MN567632	MN562124	–	MN556805
	CPC 36442^T^	–	MN562125	–	MN556806
*D.chiangraiensis*	MFLU 21–0105^T^	MZ890139	MZ890145	MZ892970	–
	KUNCC 10443	MZ890140	MZ890146	MZ892971	–
*D.chinensis*	GZCC 21–0665^T^	MZ474867	MZ474871	MZ501609	–
*D.clematidis*	MFLUCC 17–2145^T^	MT214617	MT310661	–	MT394721
	KUMCC 21–10727	OK341197	OK341184	OP413488	OP413483
*D.crassispora*	KUMCC 21–10726^T^	OK341196	OK310698	OP413479	OP413473
*D.curvularia*	KUMCC 21–10725^T^	OK341195	OK310697	OP413478	OP413472
*D.cylindricospora*	HKAS 115796^T^	OK513523	OK491122	OK524220	–
*D.dehongensis*	KUMCC 18–0090^T^	MK079662	MK085061	MK087659	–
	MFLUCC 19–0335	OK341201	OK341189	OP413491	OP413486
	MFLUCC 17–2326	OK341193	OK341183	OP413493	–
*D.effusa*	GZCC 19–0532^T^	MZ227224	MW133916	MZ206156	–
*D.euseptata*	MFUCC 20–0154^T^	MW081544	MW081539	–	MW151860
*D.euseptata*	DLUCC S2024	MW081545	MW081540	MW084994	MW084996
***D.euseptata***	**KUNCC 22–12477**	**PP068864**	**PP068487**	**PP066114**	–
*D.fasciculata*	KUMCC 19–0081^T^	MW287775	MW286501	MW396656	–
*D.fluminicola*	MFLUCC 15–0417^T^	KU376270	MF077553	–	–
*D.fusiformis*	GZCC 20–0512^T^	MZ868764	MZ868773	MZ892979	MZ892985
*D.gasaensis*	HJAUP C2034^T^	OQ942891	OQ942896	OQ944455	–
*D.guanshanensis*	HJAUP C1063^T^	OQ942898	OQ942894	OQ944452	OQ944458
*D.guizhouensis*	GZCC 21-0666^T^	MZ474869	MZ474868	MZ501610	MZ501611
*D.guttulata*	MFLUCC 16–0183^T^	MF077554	MF077543	MF135651	–
	B43	MN163016	MN163011	–	–
*D.hyalina*	MFLUCC 17–2128^T^	MZ868760	MZ868769	MZ892976	MZ892981
*D.hydei*	MFLUCC 20–0115^T^	MT742830	MT734661	–	MT767128
*D.jinghongensis*	HJAUP C2120^T^	OQ942893	OQ942897	OQ944456	–
*D.lancangjiangensis*	KUN–HKAS 112712^T^	MW879522	MW723055	–	–
*D.leonensis*	HKUCC 10822	DQ408566	–	–	DQ435089
*D.licualae*	MFLUCC 14–1163A^T^	ON650675	ON650686	ON734007	–
	MFLUCC 14–1163B^T^	ON650676	ON650687	ON734008	–
*D.lignicola*	MFLUCC 18–0198^T^	MK849797	MK828651	–	–
*D.longispora*	HFJAU 0705^T^	MH555357	MH555359	–	–
*D.longnanensis*	HJAUP C1040^T^	OQ942886	OQ942887	OQ944451	–
*D.martinii*	CGMCC 3.18651^T^	KX033566	KU999975	–	–
*D.meilingensis*	JAUCC 4727^T^	OK562396	OK562390	OK562408	–
*D.menghaiensis*	HJAUP C2045^T^	OQ942900	OQ942890	–	–
	HJAUP C2170^T^	OQ942888	OQ942899	OQ944457	OQ944461
*D.multiseptata*	MFLUCC 16–1044	MF077555	MF077544	MF135652	MF135644
*D.multiseptata*	MFLUCC 15–0609^T^	KX710140	KX710145	MF135659	–
*D.nabanheensis*	HJAUP C2003^T^	OP787877	OP787873	OP961935	–
*D.nanchangensis*	HJAUP C1074^T^	OQ942895	OQ942889	OQ944454	OQ944460
*D.neorostrata*	MFLUCC 18–0376^T^	MN163017	MN163008	–	–
*D.nonrostrata*	KUNCC 21–10730^T^	OK341198	OK310699	OP413481	OP413475
*D.obclavata*	MFLUCC 18–0329^T^	MN163010	MN163012	–	–
*D.obpyriformis*	MFLUCC 17–01694^T^	MG979764	–	MG988422	MG988415
	DLUCC 0867	MG979765	MG979757	MG988423	MG988416
***D.obpyriformis***	**KUNCC 23–13047**	**PP068865**	**PP068488**	**PP066115**	**PP066111**
*D.pachyconidia*	KUMCC 21–10724^T^	OK341194	OK310696	OP413477	OP413471
	GZCC 22–0074	ON527942	ON527934	ON533678	ON533686
***D.pachyconidia***	**KUNCC 23–13047**	**PP068866**	**PP068489**	–	**PP066112**
*D.palmarum*	MFLUCC 18–1446^T^	MK079663	MK085062	MK087660	MK087670
*D.phangngaensis*	MFLUCC 16–0857^T^	MF077556	MF077545	MF135653	–
*D.phragmiticola*	GUCC 220201^T^	OP749880	OP749887	OP749891	OP752699
	GUCC 220201^T^	OP749881	OP749888	OP749892	OP752700
*D.rayongensis*	MFLUCC 18–0415^T^	MH457137	MH457172	MH463253	MH463255
	MFLUCC 18–0417	MH457138	MH457173	MH463254	MH463256
	MFLU 19–0543	MN163010	MN513037	OP413490	OP413485
*D.rostrata*	MFLUCC 16–0969^T^	MG979766	MG979758	MG988424	MG988417
	DLUCC 0885	MG979767	MG979759	MG988425	–
*D.saprophytic*	MFLUCC 18–1238^T^	MW287780	MW286506	MW396651	MW504069
*D.septata*	GZCC 22–0078^T^	ON527947	ON527939	ON533683	ON533690
*D.sinensis*	HJAUP C2044^T^	OP787875	OP787878	OP961936	–
*D.songkhlaensis*	MFLUCC 18–1234^T^	MW287755	MW286482	MW396642	–
*D.* sp.	HKAS 112707	MZ890141	MZ890147	MZ892972	–
	HKAS 112711	MZ890142	MZ890148	MZ892973	–
***D.suae***	**CGMCC3.24262^T^**	**OQ732679**	**OQ874968**	**OR367670**	**OQ870341**
*D.suoluoensis*	MFLUCC 17–0224^T^	MF077557	MF077546	MF135654	–
	MFLUCC 17–1305	MF077558	MF077547	–	–
*D.tectonae*	MFLUCC 12–0291^T^	KX751713	KX751711	KX751710	KX751708
	MFLUCC 16–0946	MG979768	MG979760	MG988426	MG988418
	KUNCC 21–10728	OK348852	OK341185	OP413489	OP413484
	MFLUCC 15–0981	MW287763	MW286489	MW396641	–
	MFLU 20–0262	MT232719	MT232714	–	–
	KUNCC 1093	PP140788	PP140786	–	–
	KUNCC 1094	PP140789	PP140787	–	–
	MFLU 21–0106	MZ890143	MZ890149	MZ892974	–
	MFLU 21–0107	MZ890144	MZ890150	MZ892975	–
*D.tectonigena*	MFLUCC 12–0292^T^	KX751714	KX751712	–	KX751709
*D.thailandica*	MFLUCC 16–0270^T^	MH260292	MH275060	MH412767	–
*D.thysanolaenae*	KUN–HKAS 102247^T^	MK064091	MK045851	MK086031	–
*D.tropica*	GZCC 22–0076^T^	ON527943	ON527935	ON533679	ON533687
*D.verrucosa*	GZCC 20–0434^T^	MZ868762	MZ868771	MZ892977	MZ892983
*D.wuzhishanensis*	GZCC 22–0077^T^	ON527946	ON527938	ON533682	–
***D.xinpingensis***	**KUNCC 22–12669**	**OQ732680**	**OQ874969**	–	–
	**KUNCC 22–12667^T^**	**OQ732681**	**OQ874970**	**OR367671**	**OQ870340**
*D.xishuangbannaensis*	KUMCC 17–0290^T^	MH260293	MH275061	MH412768	MH412754
	GZCC 22–0079	ON527944	ON527936	ON533680	ON533688
*D.yichunensis*	HJAUP C1065^T^	OQ942892	OQ942885	OQ944453	OQ944459
*D.yongxiuensis*	JAUCC 4725^T^	OK562394	OK562388	OK562406	–
*D.yunjushanensis*	JAUCC 4723^T^	OK562398	OK562392	OK562410	–
*D.yunnanensis*	MFLUCC 20–0153^T^	MW081546	MW081541	MW084995	MW151861

Notes: The ex-type cultures are indicated using “^T^” after strain numbers; newly generated sequences are indicated in bold. “–” stands for no sequence data in GenBank.

## ﻿Results

### ﻿Phylogenetic analysis

**Figure 1.**
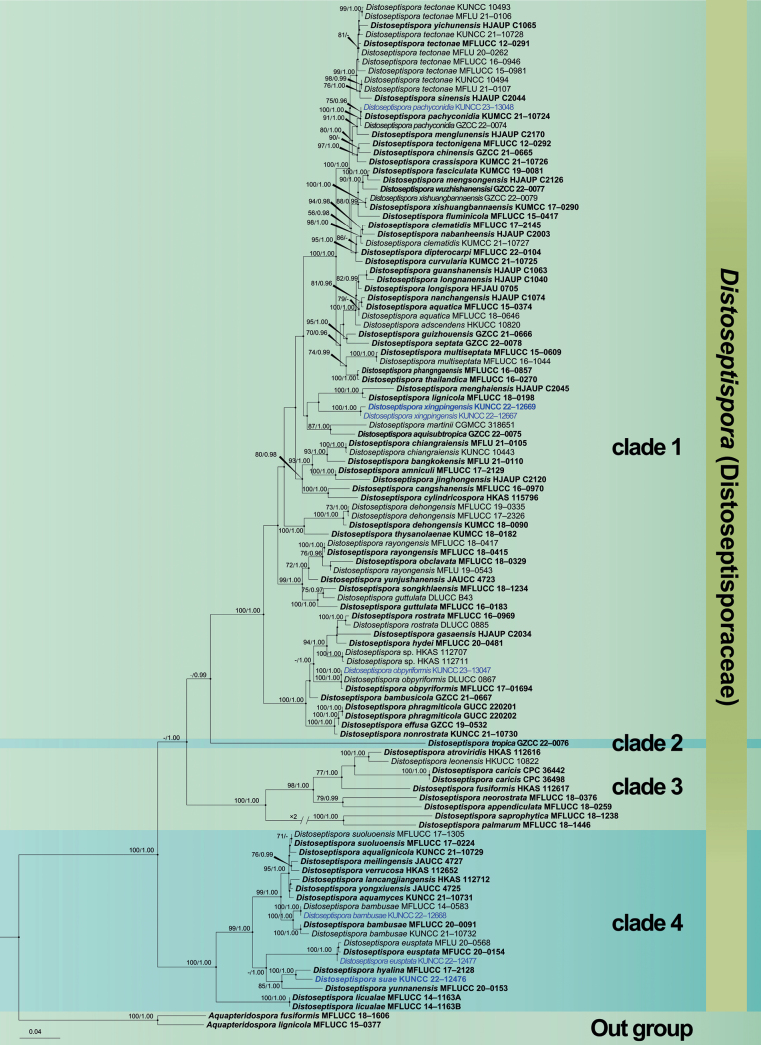
Maximum likelihood (ML) tree is based on combined LSU, ITS, *tef*1-α and *rpb*2 sequence data. Bootstrap support values with a ML greater than 65% and Bayesian posterior probabilities (PP) greater than 0.95 are given above the nodes, shown as “ML/PP”. The tree is rooted to *Aquapteridosporafusiformis* (MFLUCC 18–1606) and *A.lignicola* (MFLUCC 15–0377). New species are indicated in blue and type strains are in bold.

The dataset comprises the combined LSU, ITS, *tef*1-α and *rpb*2 sequences of 113 taxa of Distoseptisporaceae. It encompasses 3,192 characters (including gaps), with *Aquapteridosporafusiformis* (MFLUCC 18–1606) and *A.lignicola* (MFLUCC 15–0377) serving as the outgroup taxon (Fig. 1). Maximum likelihood (ML) and Bayesian analyses resulted similar topologies that were consistent spread the major clades. Likelihood of the final tree is evaluated and optimized under GAMMA. The best RAxML tree with a final likelihood value of -31566.210733 is presented (Fig. 1). The matrix contained 1,517 distinct alignment patterns, with 26.62% undetermined characters or gaps. Estimated base frequencies were as follows: A = 0.238411, C = 0.267281, G = 0.283165, T = 0.211143; substitution rates AC = 1.379525, AG = 3.509297, AT = 1.246498, CG = 0.856966, CT = 7.193589, GT = 1.000000, α = 0.242260, Tree-Length: 3.349217. Bayesian analyses generated 3,689 trees (average standard deviation of split frequencies: 0.009995) from which 2,767 were sampled after 25% of the trees were discarded as burn-in. The alignment contained a total of 1,517 unique site patterns. Bootstrap support values with a ML greater than 65%, and Bayesian posterior probabilities (PP) greater than 0.96 are given above the nodes.

Multigene phylogenetic analysis results showed that all members of *Distoseptispora* clustered into four stable clades (Fig. 1), Clade 1 contains most species of the genus with more than 50 species; Clade 2 only one species, *D.tropica*; Clade 3 contains eight species, including *D.leonensis* (M.B. Ellis) R. Zhu & H. Zhang; Clade 4 includes 13 species, and the two known teleomorph species of the genus are both in this clade. Four of our seven collections are clustered in clade 1, and the other three are clustered in clade 4. *Distoseptisporasuae* (KUNCC 22–12416) is stably aggregated in clade 4 with the teleomorph species *D.hyalina* (MFLUCC 17–2128). *D.xinpingensis* (KUNCC 22–12667 and KUNCC 22–12669) clustered as a sister clade with *D.lignicola* (MFLUCC 18–0198) in clade 1. The new collections, KUNCC 22–12668, KUNCC 22–12477, KUNCC 23–13047 and KUNCC 23–13048 clustered with *D.bambusae*, *D.euseptata*, *D.obpyriformis* and *D.pachyconidia*, respectively.

### ﻿Taxonomy

#### ﻿ Distoseptispora bambusae

Taxon classificationFungiDistoseptisporalesDistoseptisporaceae

Y.R. Sun, I.D. Goonasekara, Yong. Wang bis & K. D. Hyde, Biodiversity Data Journal 8(e53678): 6 (2020)

194B1989-2AD8-55C3-8658-18CA89F734FD

 557452

Facesoffungi Number: FoF04194

Fig. 2

##### Description.

***Saprobic*** on submerged decaying wood in a freshwater stream. **Anamorph: *Colonies*** on wood effuse, hairy, dark brown, glistening, solitary or in small group. ***Mycelium*** immersed, composed of septate, pale brown to brown hyphae, smooth-walled. ***Conidiophores*** (42–)66–103(–115) × 5–6 µm (x̄ = 84 × 6 µm, n = 20), macronematous, mononematous, solitary or in groups, erect, straight or slightly flexuous, unbranched, cylindrical, 4–6-septate, brown, rounded at the apex, slightly enlarged at the base, mooth and thin-walled. ***Conidiogenous cells*** (10–)15–22(–25) × 5–6 µm (x̄ = 19 × 5 µm, n = 20), monoblastic, terminal, determinate, subcylindrical, brown, smooth-walled. ***Conidia*** (55–)69–126(–168) × 10–12 µm (x̄ = 98 × 11 µm, n = 25), acrogenous, solitary, obclavate, rostrate, olivaceous to pale or dark brown, truncate at base, tapering towards the apex, straight or slightly curved, 7–18-euseptate, constricted at the septa, guttulate, verrucose, thick-walled. **Teleomorph**: Undetermined.

##### Culture characteristics.

Conidia germinating on PDA within 12 hrs and germ tubes produced from apex and septa of conidium. Colonies growing on PDA reaching 3–4 cm in one month at 26 °C in the dark, flocculent, fluffy, soft white to light brown mycelium from above, dark brown in the middle, light brown at the edges from below.

##### Material examined.

China, Yunnan Province, Dali City, Weishan Yi and Hui Autonomous County, 25°29′31"N, 100°06′56"E, on submerged decaying branches in a freshwater stream, 19 February 2022, Z.Q. Zhang & Q.X. Yang YJ 14-24-1 (HKAS 125826, living culture KUNCC 22–12668).

##### Notes.

**Figure 2.**
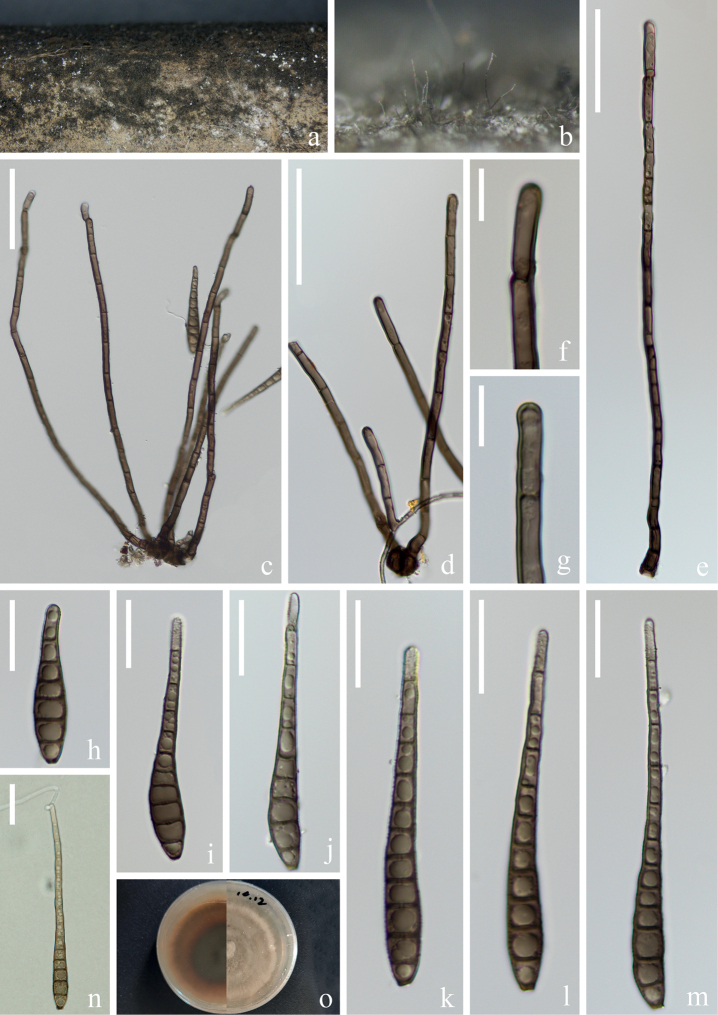
*Distoseptisporabambusae* (HKAS 125826) **a, b** colonies on woody substrates **c–e** conidiophores **f, g** conidiogenous cells **h–m** conidia **n** germinated conidium **o** culture on PDA. Scale bars: 50 μm (**c–e**); 10 μm (**f, g**); 20 μm (**h–n**).

Phylogenetic analysis showed that our new strain KUNCC 22–12668 clusters with the type strain of *Distoseptisporabambusae* (MFLUCC 14–0583) with 100% ML/1.00 PP support (Fig. 1). Furthermore, our new collection (Fig. 2) exhibits morphological characters identical to those of the type strain *Distoseptisporabambusae* (MFLUCC 14–0583). However, our collection has longer conidiophores and conidia. This observation aligns with Yang et al. (2018), suggesting that factors such as incubation time and habitat may influence the lengths of conidiophores and conidia. A comparison of the ITS, LSU, *tef*1-α and *rpb*2 sequences between our new strain KUNCC 22–12668 and the type strain (MFLUCC 14–0583) reveals only minimal base pair differences. Therefore, based on morphological evidence and phylogenetic affinity, our new strain KUNCC 22–12668 is identified as *Distoseptisporabambusae* and it is reported from freshwater habitat for the first time in Yunnan, China.

#### ﻿ Distoseptispora euseptata

Taxon classificationFungiDistoseptisporalesDistoseptisporaceae

W.L. Li, H.Y. Su & Jian K. Liu, Phytotaxa 520 (1): 80 (2021)

CAE018F6-66E7-52F0-9013-D08CAF40A5D4

 557967

Facesoffungi Number: FoF09450

Fig. 3

##### Description.

***Saprobic*** on submerged decaying wood in a freshwater stream. **Anamorph: *Colonies*** on wood effuse, brown, solitary or in small group. ***Mycelium*** mostly immersed, composed of septate, brown hyphae, smooth-walled. ***Conidiophores*** (32–)37–59(–73) × 3–4(–5) µm (x̄ = 48 × 4 µm, n = 25), macronematous, mononematous, solitary or in groups, erect, straight or slightly flexuous, branched or unbranched, cylindrical, 2–4(–5)-septate, brown, smooth-walled. ***Conidiogenous cells*** (11–)13–15(–16) × 5–6 µm (x̄ = 14 × 5 µm, n = 20), monoblastic, terminal, determinate, subcylindrical, brown, smooth-walled. ***Conidia*** (36–)52–68(–85) × (7–)8–9 µm (x̄ = 60 × 8 µm, n = 30), acrogenous, solitary, obclavate, sometimes rostrate, truncate at base, tapering towards the apex, straight or slightly curved, guttulate, brown to dark brown, 6–9(–11)-euseptate, constricted at the septa, thin and smooth-walled. **Teleomorph**: Undetermined.

**Figure 3.**
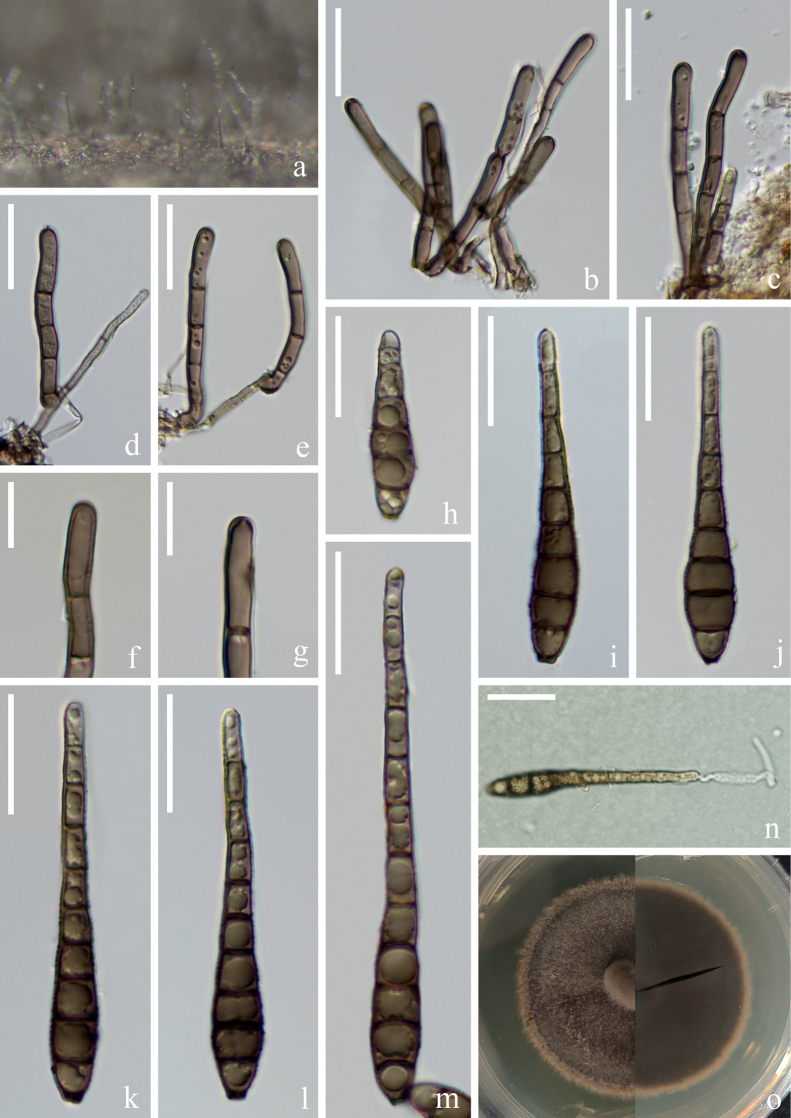
*Distoseptisporaeuseptata* (HKAS 125822) **a** colony on woody substrates **b–e** conidiophores **f, g** conidiogenous cells **h–m** conidia **n** germinated conidium **o** culture on PDA. Scale bars: 20 μm (**b–e, h–n**); 10 μm (**f, g**).

##### Culture characteristics.

Conidia germinating on PDA within 12 hrs and germ tubes produced from apex of conidium. Colonies growing on PDA reaching 4–5 cm in 20 days at 26 °C in the dark, with dense, velvety, pale brown to dark brown mycelium from above, dark brown from below.

##### Material examined.

China, Yunnan Province, Yuxi City, Xinping Yi and Dai Autonomous County, Yuanjiang River, 24°02′16"N, 101°34′05"E, on submerged decaying branches in a freshwater stream, 22 February 2022, S. Luan & W.P Wang YJ 14–49–1 (HKAS 125822, living culture KUNCC 22–12477).

##### Notes.

Polygenetic analysis revealed that our new strain, KUNCC 22–12477, clustered with two strains of *Distoseptisporaeuseptata* (MFUCC 20–0154 and MFLU 20–0568) with 100% ML/1.00 PP support (Fig. 1). A comparison of the ITS and LSU sequence between strain KUNCC 22–12477 and MFLUCC 20–0153 (from holotype) revealed 0.74% (4/537 bp, including 1 gap), 0.16% (2/1254 bp, including 2 gaps), respectively. And a comparison of the ITS, LSU and *rpb*2 sequence between strain KUNCC 22–12477 and DLUCC S2024 (from paratype) revealed 0.74% (4/537 bp, including 1 gap), 0% (0/1274 bp), and 0.23% (2/878 bp), respectively. New collection, KUNCC 22–12477, is morphologically similar to the type species in having obclavate, guttulate, euseptate conidia (Li et al. 2021). Although the conidia size and color of *D.euseptata*KUNCC 22–12477 are slightly different from the type species, and conidia size is also an important basis for distinguishing *Distoseptispora* species, some previous studies in this genus found significant differences in conidial size between different collections of the same species (Yang et al. 2018; Shen et al. 2021; Ma et al. 2022). Based on the currently limited morphological and molecular sequence data, we treat the new collection, KUNCC 22–12477, as *D.euseptata*, which was first discovered in the Red River Basin in Yunnan, China.

#### ﻿ Distoseptispora obpyriformis

Taxon classificationFungiDistoseptisporalesDistoseptisporaceae

Z.L. Luo & H.Y. Su, Mycosphere 9(3): 452 (2018)

0C6AFF50-B901-582D-8F1E-4A7226220BFB

 554290

Facesoffungi Number: FoF04194

Fig. 4

##### Description.

***Saprobic*** on submerged decaying wood in a freshwater stream. **Anamorph: *Colonies*** on wood effuse, hairy, dark brown, glistening, solitary or in small group. ***Mycelium*** immersed, composed of septate, pale brown to brown hyphae, smooth-walled. ***Conidiophores*** (42–)66–103(–115) × 5–6 µm (x̄ = 84 × 6 µm, n = 20), macronematous, mononematous, solitary or in groups, erect, straight or slightly flexuous, cylindrical, unbranched, 4–6-septate, brown, rounded at the apex, slightly enlarged at the base, smooth-walled. ***Conidiogenous cells*** (10–)15–22(–25) × 5–6 µm (x̄ = 19 × 5 µm, n = 20), monoblastic, terminal, determinate, subcylindrical, brown, smooth-walled. ***Conidia*** (55–)69–126(–168) × 10–12 µm (x̄ = 98 × 11 µm, n = 25), acrogenous, solitary, obclavate, olivaceous to pale or dark brown, truncate at base, tapering towards the apex, straight or slightly curved, constricted at the septa, 7–18-distoseptate, guttulate, thick and smooth-walled. **Teleomorph**: Undetermined.

**Figure 4.**
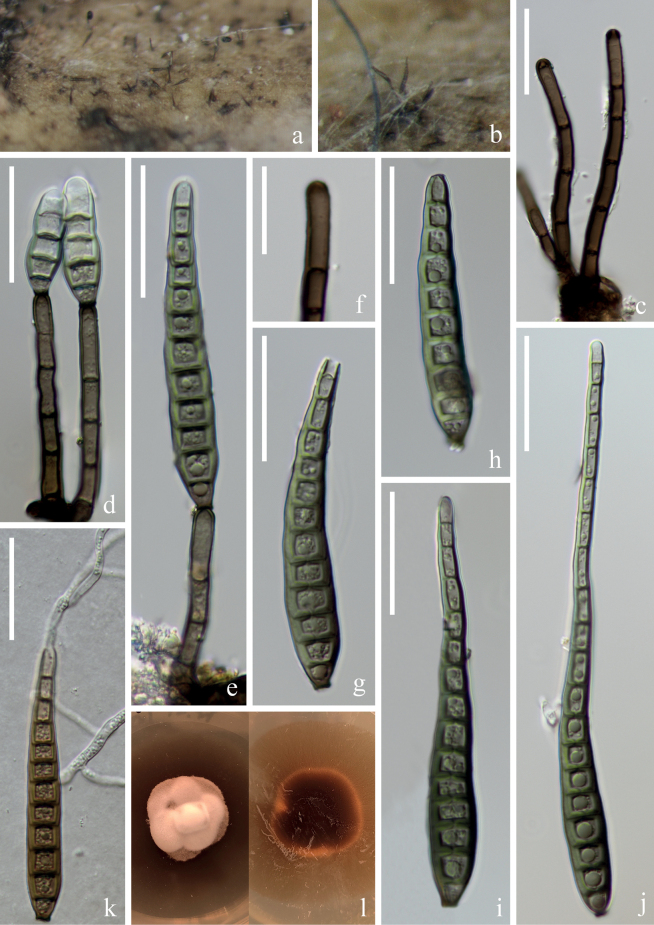
*Distoseptisporaobpyriformis* (HKAS 125823) **a, b** colonies on woody substrates **c** conidiophores **d, e** conidiophores with conidia **f** conidiogenous cell **g–j** conidia **k** germinating conidium **l** culture on PDA. Scale bars: 30 μm (**c–e, g–k**); 20 μm (**j**).

##### Culture characteristics.

Conidia germinating on PDA within 12 hrs and germ tubes produced from apex and septa of conidium. Colonies growing on PDA reaching 4–5 cm in 20 days at 26 °C in the dark, with dense, velvety, middle papillae, pale to dark brown mycelium from above; dark brown from below.

##### Material examined.

China, Yunnan Province, Dali City, Weishan Yi and Hui Autonomous County, 25°29′31"N, 100°06′56"E, on submerged decaying branches in a freshwater stream, 19 February 2022, Z.Q. Zhang & Q.X. Yang YJ 1–19–1 (HKAS 125823, living culture KUNCC 23–13047).

##### Notes.

Phylogenetic analysis revealed that our new strain KUNCC 23–13047 clustered with two strains of *Distoseptisporaobpyriformis* (MFLUCC 17–1694 (ex-type strain) and DLUCC 0867; Fig. 1). A comparison of the LSU, *tef*1-α and *rpb*2 gene of type strains between KUNCC 23–13047 (this study) and MFLUCC 17–1694 (from holotype) revealed 0% (0/1215 bp), 0.37% (3/812 bp, including 1 gaps), 0% (0/838 bp), 0.29% (3/1034 bp), respectively. Although our collection differs significantly in conidia size from the original description of *D.obpyriformis* (Luo et al. 2018a), multigene sequence data do not support this collection as a separate species. Similar results have been reported in previous studies and were found in several species in this study (Yang et al. 2018; Shen et al. 2021; Ma et al. 2022). Therefore, a new additional record of *D.obpyriformis* is reported from the Red River Basin in Yunnan, China.

#### ﻿ Distoseptispora pachyconidia

Taxon classificationFungiDistoseptisporalesDistoseptisporaceae

R. Zhu & H. Zhang, J. Fungi. 8(10): 22 (2022)

A53D4ED3-7817-5C7A-81CD-462508D90D8E

 554290

Fig. 5

##### Description.

***Saprobic*** on submerged decaying wood in a freshwater stream. **Anamorph: *Colonies*** on wood effuse, hairy, dark brown, glistening, solitary or in small group. ***Mycelium*** immersed, composed of septate, pale brown to brown hyphae, smooth-walled. ***Conidiophores*** (13–)20–36(–48) × 6–8 µm (x̄ = 28 × 7 µm, n = 30), macronematous, mononematous, solitary or in groups, erect, straight or slightly flexuous, cylindrical, unbranched, 1–3-septate, brown, rounded at the apex, slightly enlarged at the basal, smooth-walled. ***Conidiogenous cells*** 6–8 × 5–6 µm (x̄ = 7 × 5 µm, n = 25), monoblastic, terminal, determinate, subcylindrical, brown, smooth-walled. ***Conidia*** (82–)137–246(–296) × (9–)13–16 µm (x̄ = 192 × 15 µm, n = 40), acrogenous, solitary, obclavate, pale brown to brown, truncate at the base, tapering towards the apex, straight or slightly curved, 14–45-distoseptate, constricted at the septa, guttulate, thick and smooth-walled. **Teleomorph**: Undetermined.

**Figure 5.**
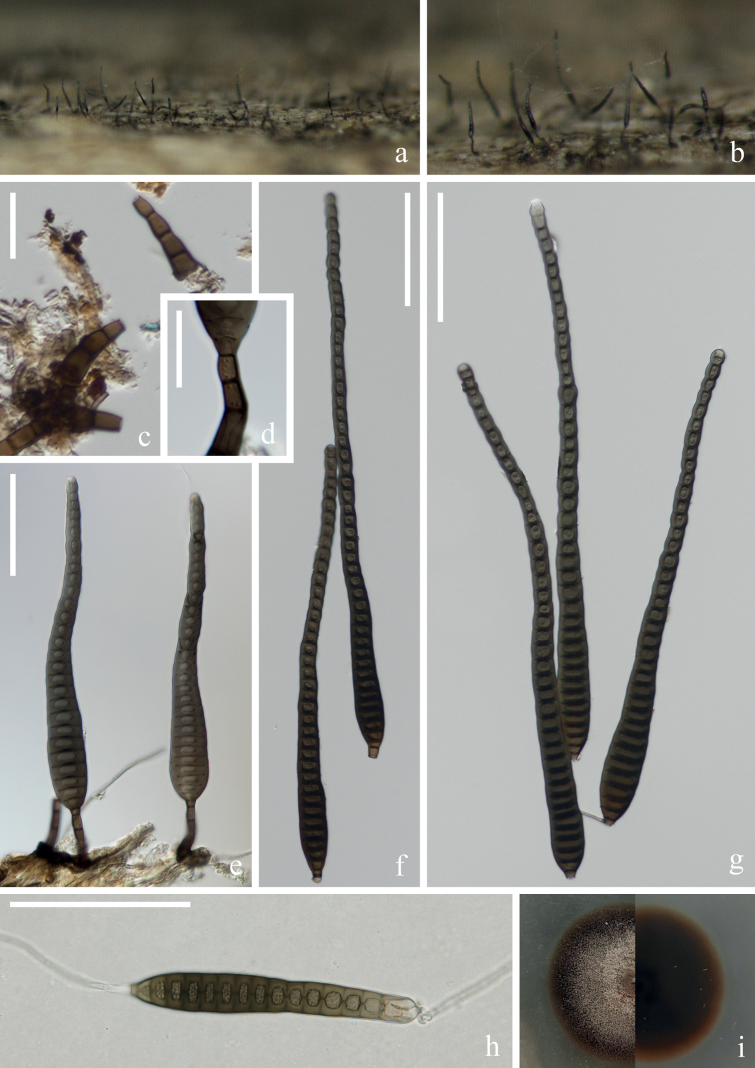
*Distoseptisporapachyconidia* (HKAS 125824) **a, b** colonies on woody substrates **c** conidiophores **e** conidiophores with conidia **d** conidiogenous cells **f, g** conidia **h** germinated conidium **i** culture on PDA. Scale bars: 20 μm (**c, d**); 60 μm (**e–h**).

##### Culture characteristics.

Conidia germinating on PDA within 12 hrs and germ tubes produced from apex and septa of conidium. Colonies growing on PDA reach 2–3 cm in one month at 26 °C in the dark, with dense, velvety, pale brown to dark brown mycelium from above; dark brown from below.

##### Material examined.

China, Yunnan Province, Honghe Hani and Yi Autonomous Prefecture, Honghe County, 23°19′32"N, 102°20′52"E, on submerged decaying branches in a freshwater stream, 23 February 2022, Z.Q. Zhang & Q.X. Yang YJ 40–30–1 (HKAS 125824, living culture KUNCC 23–13048).

##### Notes.

Phylogenetically, our new strain KUNCC 23–13048 grouped with the strains of *Distoseptisporapachyconidia* (KUMCC 21–10724 and GZCC 22–0074) with 75% ML and 0.96% PP support (Fig. 1). Pairwise comparison of ITS, LSU, *tef*1-α and *rpb*2 sequences show negligible base pair differences. As previously reported, the conidia size and color of our new collection HKAS 125824 are significantly different from those originally described for *D.pachyconidia* (137–246 µm vs. 42–136 µm; pale brown to brown vs. pale-brown with a green tinge), as well as the number of conidial septa (14–45-distoseptate vs. 8–21-distoseptate) (Yang et al. 2018; Shen et al. 2021; Ma et al. 2022). Our new collection is also slightly different from the collection described by Ma et al. (2022), especially the number of conidial septa (14–45-distoseptate vs. up to 38-distoseptate) (Ma et al. 2022). However, based on slight differences in molecular data, this collection was not sufficient to qualify as a new species, and therefore, identify this collection as *D.pachyconidia*, which was first discovered in the Red River Basin of Yunnan.

#### ﻿ Distoseptispora suae

Taxon classificationFungiDistoseptisporalesDistoseptisporaceae

H.W. Shen & Z.L. Luo sp. nov.

3A1E788B-8B8F-5668-996E-7B6C481AB2EC

Fungal Names: FN 571689

Figs 6 , 7

##### Etymology.

“suae” (Lat.) in memory of the Chinese mycologist Prof. Hong-Yan Su (4 April 1967–3 May 2022), who kindly helped the authors in many ways.

##### Description.

***Saprobic*** on submerged decaying wood in a freshwater stream. **Anamorph: *Colonies*** on wood effuse, brown, solitary or in small group. ***Mycelium*** immersed, septate, brown hyphae, smooth-walled. ***Conidiophores*** (21–)25–41(–53) × 4–5 µm (x̄ = 33 × 5 µm, n = 20), macronematous, mononematous, solitary or in groups, erect, straight or slightly flexuous, cylindrical, 1–3-septate, brown, unbranched, smooth-walled. ***Conidiogenous cells*** (11–)13–15(–16) × 5–6 µm (x̄ = 14 × 5 µm, n = 20), monoblastic, terminal, determinate, subcylindrical, brown, smooth-walled. ***Conidia*** (77–)81–101(–109) × 8–10 µm (x̄ = 91 × 9 µm, n = 30), acrogenous, solitary, obclavate to rostrate, truncate at base, tapering towards the apex, straight or slightly curved, bent at the second or third cell at the base, brown to dark brown, 3–12-euseptate, guttulate, verrucose, thin-walled. **Teleomorph**: Undetermined.

**Figure 6.**
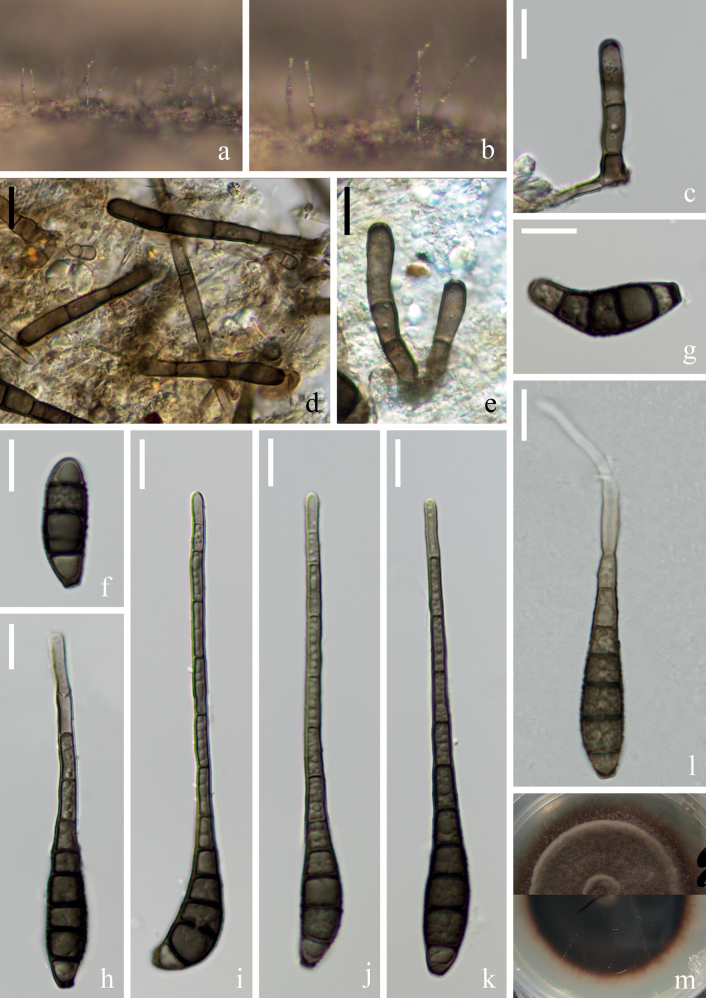
*Distoseptisporasuae* (HKAS 125819, holotype) **a, b** colonies on woody substrates **c–e** conidiophores and conidiogenous cells **f–k** conidia **l** germinated conidium **m** culture on PDA. Scale bars: 10 μm (**c–l**).

##### Culture characteristics.

**Figure 7.**
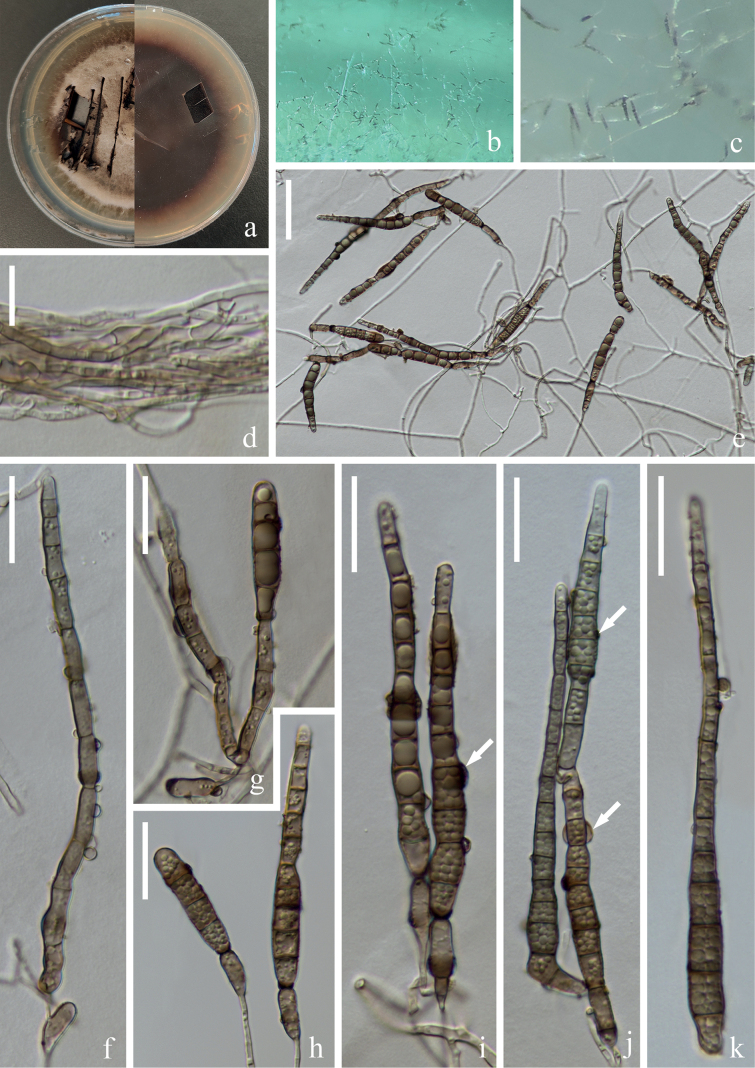
*Distoseptisporasuae* (ex-type culture KUNCC 22–12476) **a** culture on PDA, obverse (left) and reverse (right) **b, c** colonies on PDA **d** mycelium from PDA **e** mycelium, conidiophores and conidia **f** conidiophore **g–j** conidiophores with conidia (Arrow in **i, j** indicate the gelatinous sheath) **k** conidia. Scale bars: 10 μm (**d**); 40 μm (**e**); 20 μm (**f–k**).

Conidia germinating on PDA within 12 hrs and germ tubes produced from the apex. Colonies growing on PDA reaching 4–5 cm in one month at 26 °C in the dark, with dense, velvety, brown to dark brown mycelium from above; dark brown from below. Sporulation on PDA after two months, ***Mycelium*** hyaline to brown, septate, branched, smooth-walled. ***Conidiophores*** (15–)16–56(–110) × 4–6 µm (x̄ = 36 × 5 µm, n = 30), usually form at the end of the hyphae, cylindrical, straight or slightly curved, yellowish brown to olivaceous-brown, septate. ***Conidiogenous cells*** (9–)11–14(–16) × 4–5 µm (x̄ = 12 × 5 µm, n = 30) monoblastic, terminal, determinate, cylindrical, brown, sometimes reduce conidiophores. ***Conidia*** (31–)47–90(–124) × 6–8 µm (x̄ = 68 × 7 µm, n = 40) acrogenous, obclavate, elongated, truncate at base, straight or slightly curved, brown, euseptate, thin-wall, sometimes with a gelatinous sheath around the septum (Fig. 7).

##### Material examined.

China, Yunnan Province, Yuxi City, Xinping Yi and Dai Autonomous County, Yuanjiang River, 24°02′16"N, 101°34′05"E, on submerged decaying branches in a freshwater stream, 22 February 2022, H.W. Shen & Q.X. Yang YJ 14–35–2 (HKAS: 125819, holotype, ex-type, CGMCC3.24262 = KUNCC 22–12476).

##### Notes.

*Distoseptisporasuae* clusters with *D.hyalina* (MFLU 21–0137) with 100% ML/1.00 PP support whereas *D.yunnanensis* (MFLUCC 20–0153) state in a basal lineage (Fig. 1). A comparison of the LSU, ITS, *tef*1-α and *rpb*2 nucleotide bases between *D.suae* and *D.hyalina* revealed differences of 8 bp (8/832, including one gap), 11 bp (11/540, including 3 gaps), 21 bp (21/934), and 54 bp (54/1087) sequence similarity, respectively. Morphologically, *D.suae* resembles other species in the genus with its euseptate structure, characterized by acrogenous, solitary, obclavate to rostrate conidia. *D.hyalina*, *D.suae* and *D.yunnanensis* cluster in a stable lineage (Fig. 1). Since only teleomorphs were found in *D.hyalina* (Yang et al. 2021), and only anamorphs were found in *D.suae* (this study), the morphological characteristics of *D.suae* and *D.yunnanensis* were compared here. *D.suae* can be distinguished from *D.yunnanensis* by its shorter conidiophores (25–41(–53) µm vs. 131–175 µm) and guttulate, verrucose conidia (Li et al. 2021). Based on phylogenetic analysis and morphological evidence, following the guidelines of Jeewon and Hyde (2016), we therefore introduce *D.suae* as a new species.

#### ﻿ Distoseptispora xinpingensis

Taxon classificationFungiDistoseptisporalesDistoseptisporaceae

H.W. Shen & Z.L. Luo sp. nov.

E38A2BAD-0E84-52A1-94FC-3CCE8DA14E70

Fungal Names: FN 571753

Fig. 8

##### Etymology.

“*xinpingensis*” refers to the Xinping Yi and Dai Autonomous County, Yunnan Province, China, where the species was collected.

##### Description.

***Saprobic*** on submerged decaying wood in a freshwater stream. **Anamorph: *Colonies*** on wood effuse, brown to dark brown, solitary or gregarious. ***Mycelium*** immersed, composed of septate, hyaline to brown hyphae, smooth-walled. ***Conidiophores*** (97–)105–149(–175) × 4–5 µm (x̄ = 127 × 5 µm, n = 40), macronematous, mononematous, solitary or in groups, erect, straight or slightly flexuous, cylindrical, brown, unbranched, slightly paler at the apical cell, slightly enlarged at the base, septate, smooth-walled. ***Conidiogenous cells*** (7–)13–23(–25) × 4–5 µm (x̄ = 18 × 4 µm, n = 30), mono- or poly- blastic, terminal, determinate, subcylindrical, pale brown, smooth-walled. ***Conidia*** (95–)107–139(–155) × (7–)8–9(–10) µm (x̄ = 123 × 8 µm, n = 40), acrogenous, solitary, obclavate, truncate at base, tapering towards the apex, straight or slightly curved, brown, 8–12-euseptate, smooth, thin-wall, sometimes a second conidium proliferates at the top of the conidia. **Teleomorph**: Undetermined.

**Figure 8.**
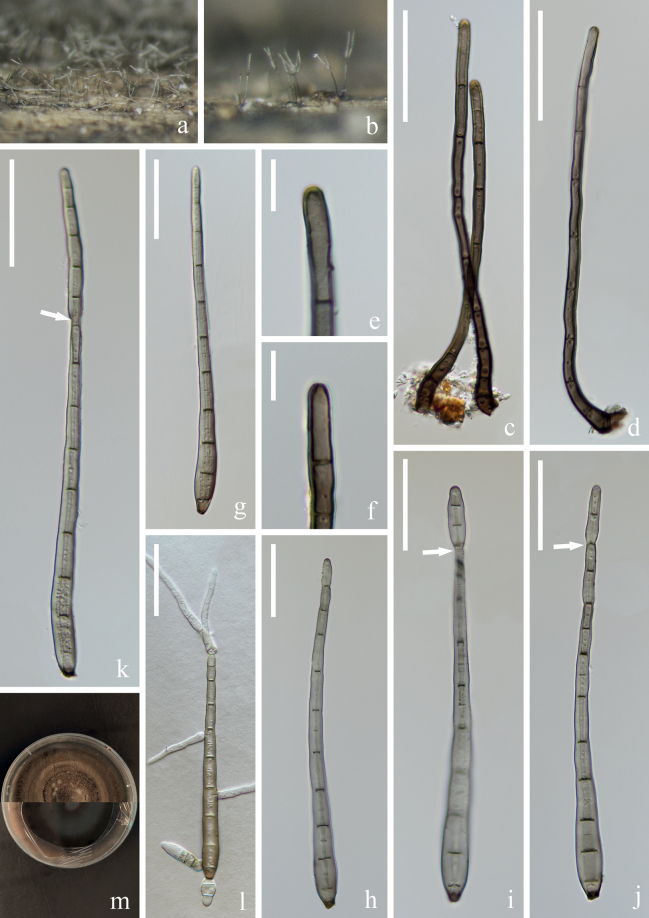
*Distoseptisporaxinpingensis* (HKAS 125818, holotype) **a, b** colonies on woody substrates **c, d** conidiophores **e, f** conidiogenous cells **g–k** conidia (Arrow in **i–k** indicate proliferating conidia) **l** germinating conidium **m** culture on PDA. Scale bars: 40 μm (**c, d**); 10 μm (**e, f**); 30 μm (**g–l**).

##### Culture characteristics.

Conidia germinating on PDA within 24 hrs and swollen germ tubes produced from both ends and some septate. Colonies growing on PDA reaching 2–3 cm in two weeks at 26 °C in the dark, with dense, velvety, dark brown mycelium on the surface; in reverse brown to dark brown with entire margin.

##### Material examined.

China, Yunnan Province, Yuxi City, Xinping Yi and Dai Autonomous County, Yuanjiang River, 23°48′12"N, 101°47′21"E, on submerged decaying branch in a freshwater stream, 22 February 2022, S. Luan & W.P Wang YJ 17–2–2 (HKAS: 125818, holotype), ex-type, KUNCC 22–12667; ibid, 23°48′12"N, 101°47′21"E, on submerged decaying branches in a freshwater stream, 22 February 2022, H.W. Shen & Z.Q. Zhang YJ 17–5–2 (HKAS: 125821, paratype), ex-paratype, KUNCC 22–12669.

##### Notes.

Phylogenetic analysis showed that the two strains of *Distoseptisporaxinpingensis* (KUNCC 22–12669 and KUNCC 22–12667) clustered together and formed a sister clade to *D.lignicola* (MFLUCC 18–0198) and *D.menghaiensis* (HJAUP C2045) with low support (Fig. 1). Based on a megablast search of NCBIs GenBank nucleotide database, the best matching result for ITS sequence of KUNCC 22–12667 is *Distoseptispora* sp. (isolate SICAUCC 22–0049, sequence ID: ON228626; identities: 499/516 (97%), 4 gaps); the best matching result of LSU sequence is *D.lignicola* (strain MFLUCC 18–0198, sequence ID: MK849797; identities: 1223/1245 (98%), no gap); the best matching result of *rpb*2 sequence is *D.bambusae* (voucher MFLU 17–1653, sequence ID: MT232882; identities: 1047/1047 (100%), no gap); the best matching result of *tef*1-α sequence is *D.mengsongensis* (strain HJAUP C2126, sequence ID: OP961937; identities: 866/916 (95%), no gap). *Distoseptisporaxinpingensis* conforms to the generic concept of *Distoseptispora* (Su et al. 2016; Yang et al. 2018, 2021; Luo et al. 2019; Zhang et al. 2022). The morphological comparison between *Distoseptisporaxinpingensis* and the closely related *D.lignicola* and *D.menghaiensis* shows that *D.xinpingensis* has longer conidiophores (105–149 µm vs. 84–124 µm) and conidia (107–139 µm *vs.* 60–108 µm), and with more conidial septate (8–12 *vs.* 5–9) than *D.lignicola*. *Distoseptisporaxinpingensis* can be distinguished from *D.menghaiensis* by its longer conidiophores (105–149 µm *vs.* 45.7–82.9 µm) and conidia (107–139 µm *vs.* 35.7–48.6 µm), as well as conidial septation (8–12-euseptate *vs.* 4–8-distoseptate) (Hu et al. 2023). Given the morphological distinctions and evidence from phylogenetic analysis, we introduce *Distoseptisporaxinpingensis* as a new species from the Red River Basin in Yunnan, China.

## ﻿Discussion

Systematic research on lignicolous freshwater fungi is ongoing in the Red River Basin. Seven distoseptispora-like species were discovered from submerged decaying wood. Based on multigene phylogenetic analysis and morphological studies, six *Distoseptispora* species were identified, *D.suae* and *D.xinpingensis* were introduced as new species with their unique morphology and phylogenetic placement. Previously introduced species, *D.bambusae*, *D.euseptata*, *D.obpyriformis* and *D.pachyconidia* were reported in the watershed for the first time. The Red River Basin may contain more interesting, particular, and undiscovered freshwater fungal species, as no studies have been reported on yet.

In the past seven years, more than 70 *Distoseptispora* species have been introduced based on morphological and molecular evidence. These species grow as saprophytes on a variety of decaying wood debris in tropical and subtropical freshwater and terrestrial habitats (Index Fungorum database; Hu et al. 2023). 45 species have been reported on submerged bamboo stems and unknown wood debris in freshwater habitats, and 23 species have been reported on dead leaves, branches, and stems of various plants in terrestrial habitats, such as palms (Hyde et al. 2019), bamboo (Monkai et al. 2020), grasses (Hyde et al. 2023), and unknown broad-leaved trees (Hu et al. 2023), etc., and five species have been reported in both terrestrial and freshwater habitats (Hyde et al. 2016; Tibpromma et al. 2018; Phookamsak et al. 2019; Phukhamsakda et al. 2020; Sun et al. 2020; Shen et al. 2021; Ma et al. 2022; Zhang et al. 2022). China and Thailand are the countries that contribute the most *Distoseptispora* species, with 50 species reported in China and 25 species reported in Thailand.

Species of *Distoseptispora* are usually distinguished based on phylogenetic analysis combined with the morphological characteristics of conidiophores and conidia (Su et al. 2016; Monkai et al. 2020; Zhang et al. 2022). Important morphological characteristics are the color, shape, size, septation type (distoseptate/euseptate) and number of conidia, as well as the length of the conidiophores (Zhang et al. 2022). Phylogenetic studies of *Distoseptispora* are usually based on ITS, LSU, *tef*1-α, and *rpb*2 gene loci, and currently, all type species are well resolved on the phylogenetic tree (Hu et al. 2023; this study). Several previous studies have shown that new specimens of some species of *Distoseptispora* are significantly different in morphology from original descriptions, especially in the color and size of the conidia (Yang et al. 2018; Luo et al. 2019; Shen et al. 2021; Ma et al. 2022). These new specimens are usually collected from different habitats from the type specimens, and sometimes from the same habitat (Yang et al. 2018; Luo et al. 2019; Shen et al. 2021; Ma et al. 2022). However, the ITS, LSU, *tef*1-α and *rpb*2 sequences of these new specimens are not significantly different from the type specimens (Yang et al. 2018; Shen et al. 2021; Ma et al. 2022). Habitat and incubation time may affect the size of conidia, but this has not yet been determined and needs to be resolved in future studies (Yang et al. 2018; Shen et al. 2021; this study). Additionally, the brand and photography mode of the compound microscope may affect the color of the conidia. Of course, another possibility is that the four loci currently used to construct the phylogenetic analysis are not enough to provide more information to explain the morphological differences; combining more loci or a whole-gene phylogenetic study may explain these morphological differences.

The multigene phylogeny indicates that members of *Distoseptispora* are distributed in four distinct clades. However, there are no pronounced morphological differences sufficient to separate them (Zhang et al. 2022). Morphologically, *Distoseptisporamartinii* stands apart from other species within the genus due to its ellipsoid, oblate or subglobose and muriform conidia. These characteristics align more closely with the general concept of *Junewangia*. Therefore, the phylogenetic placement of *D.martinii* requires further examination through nucleotide base sequence analysis. In subsequent studies, the culture of *D.martinii* (CGMCC 3.18651) could be encouraged to sporulate to determine if similar conidiophores and conidia are produced. Notably, in our study, the ex-type strain of *D.suae* (KUNCC 22–12476) produced conidiophores and conidia that mirrored those observed on the natural substrate, confirming this approach as promising.

## Supplementary Material

XML Treatment for Distoseptispora bambusae

XML Treatment for Distoseptispora euseptata

XML Treatment for Distoseptispora obpyriformis

XML Treatment for Distoseptispora pachyconidia

XML Treatment for Distoseptispora suae

XML Treatment for Distoseptispora xinpingensis

## ﻿Additional information

### Author ORCIDs

Hong-Wei Shen https://orcid.org/0000-0003-2508-1970

Dan-Feng Bao https://orcid.org/0000-0002-5697-4280

Saranyaphat Boonmee https://orcid.org/0000-0001-5202-2955

Yong-Zhong Lu https://orcid.org/0000-0002-1033-5782

Xi-Jun Su https://orcid.org/0000-0002-6357-7750

Yun-Xia Li https://orcid.org/0009-0007-5645-8861

Zong-Long Luo https://orcid.org/0000-0001-7307-4885

### Data availability

All of the data that support the findings of this study are available in the main text.
